# Prenatal diagnosis of complete maternal uniparental isodisomy of chromosome 4 in a fetus without congenital abnormality or inherited disease-associated variations

**DOI:** 10.1186/s13039-015-0190-z

**Published:** 2015-11-04

**Authors:** WeiQiang Liu, HuiMin Zhang, Jian Wang, GuoJiu Yu, WenJun Qiu, ZhiHua Li, Min Chen, Kwong Wai Choy, XiaoFang Sun

**Affiliations:** Key Laboratory of Reproduction and Genetics of Guangdong Higher Education Institutes, Key Laboratory for Major Obstetric Diseases of Guangdong Province, Third Affiliated Hospital of Guangzhou Medical University, Guangzhou, 510150 P. R. China; Department of Obstetrics and Gynaecology, The Chinese University of Hong Kong, Hong Kong, P. R. China; Department of Laboratory Medicine, Shanghai Children’s Medical Center, Shanghai Jiao Tong University School of Medicine, Shanghai, 200127 P. R. China; Department of Prenatal Diagnosis and Fetal Medical, Third Affiliated Hospital of Guangzhou Medical University, Guangzhou, 510150 P. R. China

**Keywords:** Uniparental isodisomy, Chromosome 4, Chromosomal microarray analysis, Whole exome sequencing, Ultrasound

## Abstract

**Background:**

The prenatal diagnosis of subjects with complete uniparental isodisomy of chromosome 4 (iUPD4) has rarely been reported and poses a great challenge for genetic counseling. In this study, a prenatal case with a high (1 in 58) risk of Down syndrome was diagnosed with iUPD4 by combined chromosomal microarray analysis (CMA), whole exome sequencing (WES) and ultrasound morphology scan.

**Results:**

By CMA, a pathogenic copy number variant was not detected; however, a complete maternal iUPD4 was identified in this fetus after analyzing the parental genotype results. To detect potentially autosomal recessive variants, WES was performed. Two missense and two frameshift variants were identified but were predicted with uncertain significance; none of the mutations were definitively associated with congenital abnormality or inherited disease. In addition, a detailed ultrasound morphology scan did not identify any structural abnormalities, facial dysmorphisms or intrauterine growth restriction. The family history was unremarkable. The couple was counseled with the prenatal diagnostic results, and they opted to give birth to the child. No phenotypic abnormalities were observed in this child after the first year of life.

**Conclusion:**

This study provides further evidence that iUPD4 can result in a healthy live birth and demonstrates that the combined use of CMA, WES and ultrasound technology provides additional information for the prenatal diagnosis and clinical management of rare UPD events.

**Electronic supplementary material:**

The online version of this article (doi:10.1186/s13039-015-0190-z) contains supplementary material, which is available to authorized users.

## Background

Uniparental disomy (UPD) is defined as an inheritance of both homologous chromosomes from a single parent without contribution from the other parent [1]. Based on different mechanisms and different origins of the disomic chromosome, UPD can present as maternal UPD, paternal UPD, uniparental heterodisomy (hUPD, two different chromosomal homologs inherited from the same parent), uniparental isodisomy (iUPD, two identical copies of a single homolog), whole-chromosome UPD and segmental UPD [2–4].

The incidence of UPD is estimated to be approximately 1:3500 live births [5]. The pathogenesis of UPD is always associated with imprinting disorders or the unmasking of homozygous mutations in iUPD, which can trigger autosomal recessive diseases [6–8]. To date, the clinically relevant UPD phenotypes that have been definitively associated with imprinting disorders are limited to regions of 6q24, 7p11.2-p12, 7p32.2, 11p15.5, 14q32.2, 15q11-q13 and 20q13.3 [9–13]. Therefore, genetic counseling is difficult, especially counseling for prenatal diagnoses and for those exhibiting UPD on chromosomal regions that are not listed above. In addition, although more than 100 imprinted genes that have been identified in the human genome (http://www.geneimprint.com/), the understanding of how most of the imprinted genes function is limited. Tissue-specific imprinting and controversial reports of some imprinted genes adds to the complexity for genetic counseling [14–16]. Furthermore, merely relying on ultrasonography data for prenatal genetic counseling makes it challenging to precisely predict which homozygous variations will “activate” recessive mutation events or trigger autosomal recessive disorders in a fetus without obvious dysmorphisms.

Although more than 2,800 UPD cases have been reported in the literature (http://upd-tl.com/upd.html), a case of complete maternal iUPD of chromosome 4 is very rare, and a prenatal subject with UPD4 has only been reported once. Currently, there is only one reported case of prenatal hUPD4 [17], three postnatal reported cases of complete maternal iUPD4 [18–20] and fewer than 10 postnatal reported cases of segmental maternal iUPD or mixed hUPD and iUPD of chromosome 4 [21–23]. Among these reported cases, most of the clinical phenotypes presented were due to the homozygosity of a recessive mutation rather than aberrant imprinting, suggesting that important maternally imprinted genes are not located on chromosome 4.

Chromosomal microarray analysis (CMA) has been used for the prenatal diagnosis of fetuses that present with ultrasound anomalies, and single-nucleotide polymorphism (SNP)-based CMA has further facilitated the detection of iUPD or identical by descent (IBD) segments, as well as copy number variations [24–26]. Whole exome sequencing (WES) is a powerful tool to search for potential pathogenic variants located in an iUPD region [27]. In this study, a complete maternal iUPD of chromosome 4 was identified using a SNP-based CMA from a fetal sample, and subsequently WES was used to identify potential pathogenic variants. None of the sequence variants that were identified using WES were associated with congenital abnormality or inherited disease, and no structural abnormalities, facial dysmorphisms or intrauterine growth restriction (IUGR) was observed in this fetus using detailed ultrasound scanning. The couple was counseled with the prenatal diagnostic results and was additionally informed that an imprinting effect associated with iUPD4 cannot be excluded. The couple opted to give birth to the child. A healthy boy was born by vaginal delivery at 40 weeks with a birth weight of 2.70 kg, length of 49 cm, head circumference of 33 cm and Apgar scores of 10 each at 1, 5 and 10 min post-delivery.

## Results

### Timeframe of gestational weeks for different prenatal analyses

A brief outline of the timeframe of gestational weeks for different prenatal diagnoses is listed in Additional file 1: Table S1.

### Identifying CNVs and UPD events with SNP-based CMA

**Fig. 1:**
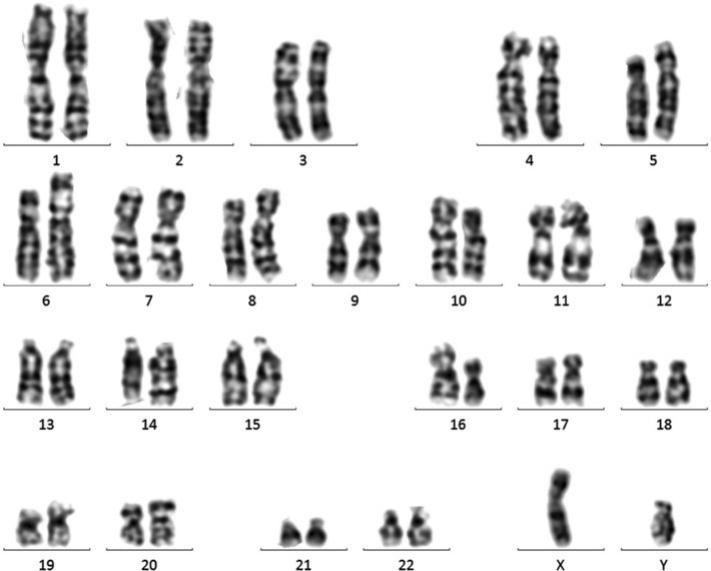
Karyotype analysis of the fetus. The fetal amniotic fluid sample showed a normal 46,XY karyotype, and no structural abnormalities or small markers were observed after counting 20 metaphase cells

**Fig. 2:**
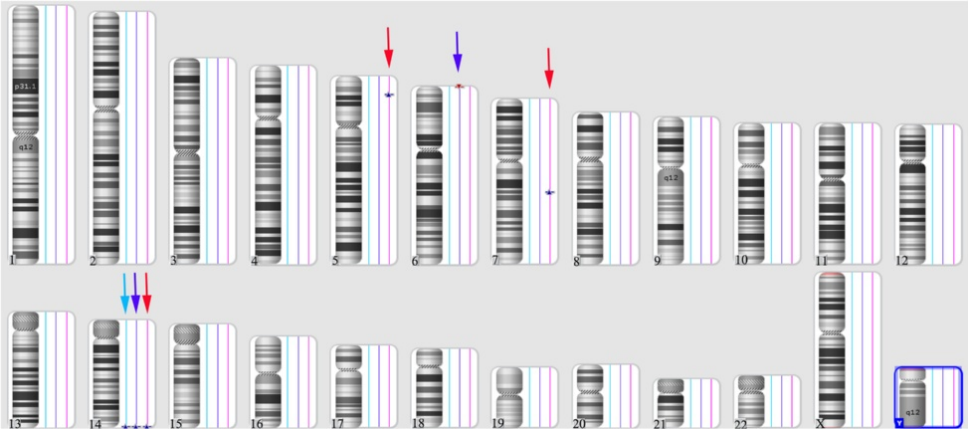
Identification of copy number variations. ChAS software indicated a single deletion (red triangle) and a single duplication of CNVs (blue triangle) in the fetal sample (blue arrow). Three duplicated CNVs were observed in the mother (red arrow), whereas only a single duplicated CNV was observed in the father (light blue arrow)

Karyotype testing of the fetal amniotic fluid sample revealed a normal result (46,XY) (Fig. 1). For CNV analysis, only a single deletion and a single duplication were identified in the fetal DNA sample, three duplications were found in the mother, and only a single CNV duplication was observed in the father (Fig. 2, Table 1.). All of the identified CNVs were evaluated and have previously been reported as benign in public databases.

**Table 1:** CNVs identified in the iUPD4 case

Subject	CNVs region	CNV State	Type	Sizes	Genes within CNVs	Function
Fetus	chr6:254,253-320,842	1	Loss	67Kb	DUSP22	Benign
	chr14:106,251,069-106,736,227	3	Gain	485Kb	KIAA0125	Benign
					ADAM6	
Father	chr14: 106,167,581-106,769,864	3	Gain	602Kb	KIAA0125	Benign
					ADAM6	
					LINC00226	
Mother	chr14:106,167,581-106,766,782	3	Gain	599Kb	KIAA0125	Benign
					ADAM6	
					LINC00226	
	chr5:17,398,797-17,701,556	3	Gain	303Kb	/	Benign
	chr7:89,367,271-90,601,186	3	Gain	314Kb	DPY19L2P4	Benign
					STEAP1, STEAP2 et al.	

### Identification of UPD events

**Fig. 3:**
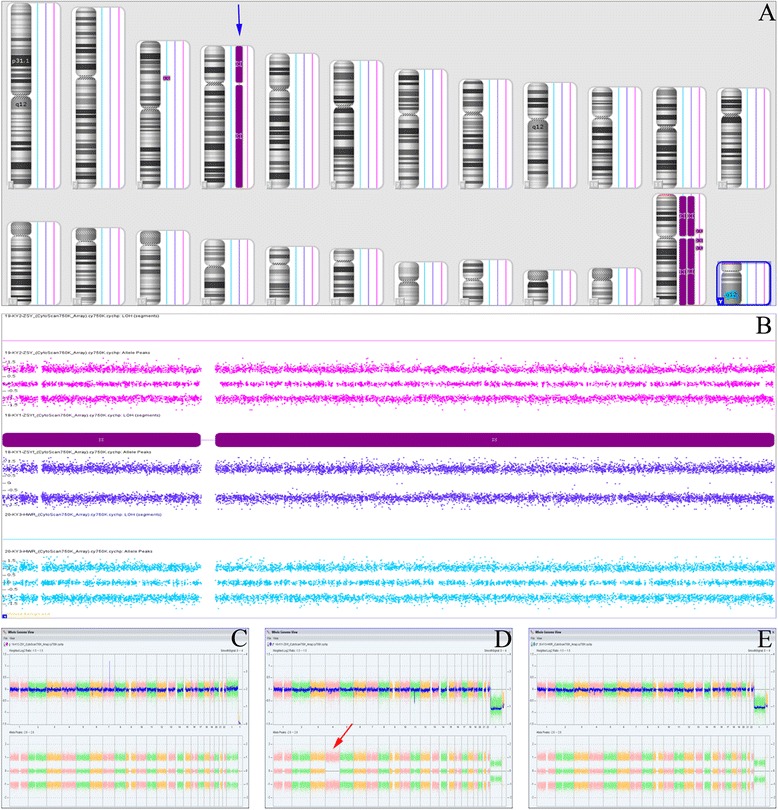
Complete maternal uniparental isodisomy of chromosome 4. **a** ChAS revealed a complete iUPD of chromosome 4 in the fetus (blue line) by showing an absence of heterozygosity (AOH) across the entire chromosome (purple rectangle, blue arrow). The results of an AOH analysis were normal for his mother (red line) and his father (light blue line). The purple rectangle on the X chromosome indicates the hemizygous state in the male samples. **b** Three allelic lines indicate the heterozygous state of the mother (red line) and father (light blue line), whereas the two allelic lines indicate that an AOH occurred for a whole chromosome of the fetus (blue line). **c-e** A whole chromosome view clearly shows the copy neutral AOH on chromosome 4 in the fetus (red arrow) and a normal chromosome 4 in his parents

**Fig. 4:**
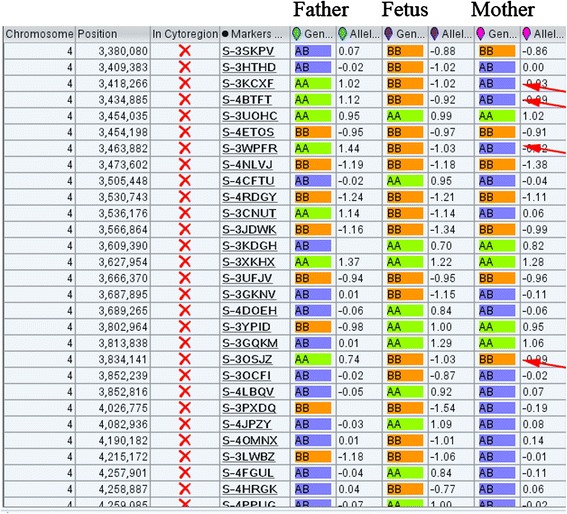
ChAS software directly indicates the origin of the isodisomic UPD. After comparing the genotyping results between the fetus and his parents, ChAS software clearly indicated that the UPD of chromosome 4 in the fetus originated from the mother. At position chr4:3,418,266, the genotyping was BB, AA and AB in the fetus, his father and his mother, respectively, which indicates that the B allele in the fetus was duplicated from his mother. There are many different alleles that can be used to identify the origin of chromosome 4 in the fetus (red arrows)

Although no pathogenic CNVs were identified in either the fetus or his parents, the SNP array indicated a complete iUPD of chromosome 4 in the fetus based on the absence of heterozygosity (AOH) across the entire chromosome (Fig. 3a and b). A whole genome view using the ChAS software program clearly identified a 187-Mb isodisomic UPD in the entire chromosome 4 region of the fetus but detected no abnormalities in his parents (Fig. 3c-e). In addition, the software directly indicated that the isodisomic UPD was of maternal origin (Fig. 4), and this was validated by comparing 13,201 SNPs on chromosome 4 derived from the CMA results (from rs12511220 at chr4:75174 to rs7686607 at chr4:190921709) between the fetus and his parents (Additional file 2).

The Genomic Oligoarray and SNP array evaluation tool located a total of 1,472 genes on chromosome 4. Among them, 552 were OMIM genes, and 131 genes were disease associated, including 63 autosomal recessive genes and 44 autosomal dominant genes (Additional file 3).

### Whole exome sequencing analysis

WES was performed to investigate whether there were any SNVs or InDels located in the iUPD regions. WES yielded a total of 47,224,320 mapped reads with 93.62 % on target. The total assigned amplicon reads were 44,210,426. Average reads per amplicon was 150.4, with 45.66 % of targets having 100X coverage and 89.41 % having 20X coverage. The variant call format (VCF) files were submitted to the wANNOVAR web server (http://wannovar.usc.edu/) for identification and filtering of the SNVs. We identified 51,235 variants, including 47,363 single nucleotide variations, 224 multiple nucleotide variations (MNV) and 3,648 InDels. Among these variants, there were 1,343 homozygous SNVs, 3 homozygous MNVs and 65 homozygous InDels that were identified on chromosome 4 (Table 2).

**Table 2:** Summary of variants detected via WES

Type of Variants	Total Variants	Variants on Chromosome 4
Homozygous SNVs	20003	1343
Homozygous MNVs	20	3
Homozygous INDELs	1117	65
Heterozygous SNVs	27360	139^a^
Heterozygous MNVs	204	15^a^
Heterozygous INDELs	2531	61^a^

^a^All heterozygosity variants with low quality are likely representative of sequencing errors

For variant filtering, the variants observed with an allele frequency greater than or equal to 3.0 % of the genomes in the 1000 genomes project were excluded and those variants associated with a phenotype variant were subjected to further analysis. After filtering using Ingenuity software, 2 frameshift and 2 nonsynonymous variants located on chromosome 4 were identified (Table 3). Although these variants were predicted to have damaging or possibly damaging functions, none of them were OMIM disease genes, and none of those variants were definitely associated with congenital abnormality or inherited disease. Therefore, in this study, all of the four variants were classified as Variants of Uncertain Significance (VOUS).

**Table 3:** Four rare homozygous variants on chromosome 4

Gene Symbol	Position	Variation Type	Transcript Variant	Protein Variant	SIFT Function Prediction	PolyPhen-2 Function Prediction	Classification
KIAA1211	57180473	Deletion	c.805delC	p.L269fsX13			Uncertain Significance
MMRN1	90855967	SNV	c.1136A > T	p.K379I	Damaging	Possibly Damaging	Uncertain Significance
SLC25A31	128665845	SNV	c.251G > A	p.R84H	Damaging	Possibly Damaging	Uncertain Significance
NPY5R	164272419	Deletion	c.997delG	p.V333fsX7			Uncertain Significance

### Confirmation of identified variants

**Fig. 5:**
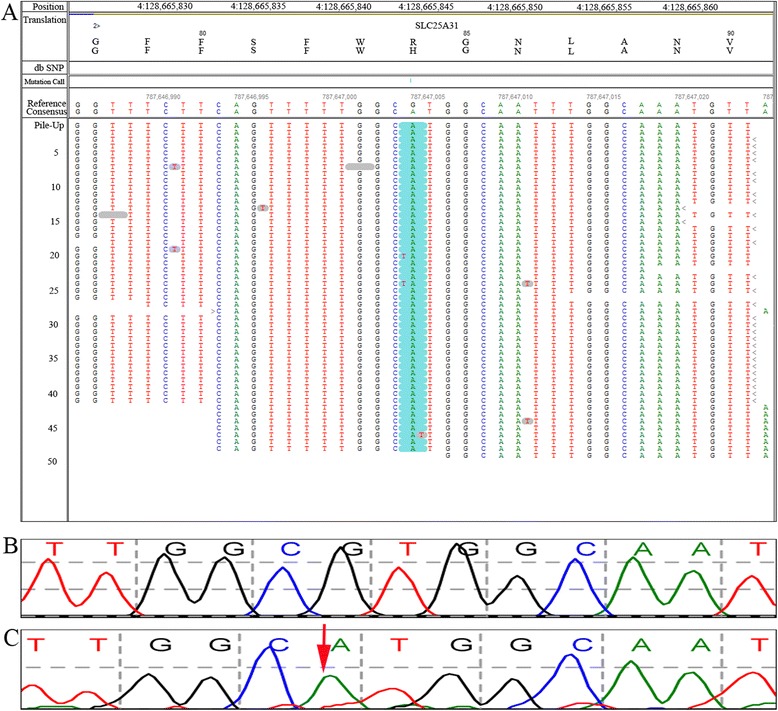
Rare variants identified via WES. **a** A homozygous variant was identified in the *SLC25A31* gene (c.251G > A, p.R84H) via the NGS method. **b** Sanger sequencing reference of *SLC25A31*. **c** The c.251G > A mutation (red arrow) was validated by Sanger sequencing

More than 99 % of the SNPs identified in the WES analysis coincided with the results derived from the CMA (data not shown). All four VOUS variants were successfully validated by Sanger sequencing (Fig. 5).

### Ultrasound morphology scans

**Fig. 6:**
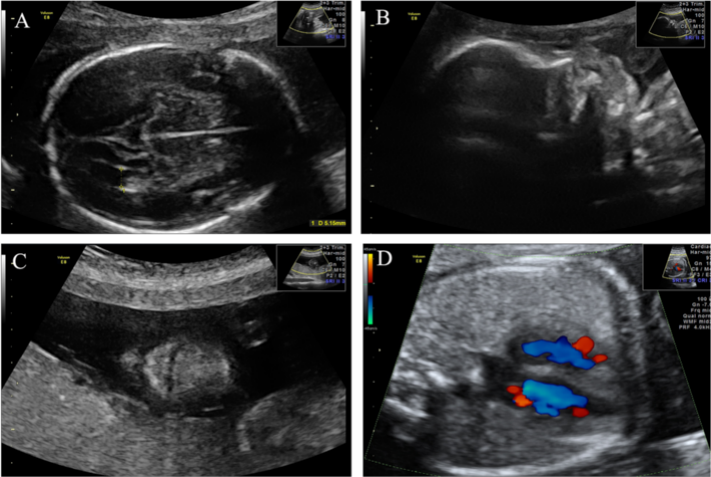
Detailed screening by ultrasound. No facial cleft or other structural abnormalities were observed in this fetus. Detailed ultrasound screening at 25 weeks of gestation show (**a**) a normal lateral ventricle view; (**b**) nasal bone view; (**c**) palate and lip view; and (**d**) apical four chamber heart view

Because no pathogenic variants were identified in the fetus carrying iUPD4, a detailed ultrasound screening was performed to monitor the fetal development. Malformations detectable with ultrasound, structural abnormalities, facial dysmorphisms and intrauterine growth restrictions (IUGRs) were not observed in the fetus (Fig. 6). In addition, no other clinical phenotype was observed by the pediatrician during a follow-up period up to the first year after birth with a weight of 9.50 kg (50th), length of 74.1 cm (50th) and head circumstance of 45.5 cm (25th).

## Discussion

The widespread use of SNP-based CMA technology has facilitated the detection of UPD events [28]. SNP-based CMA is a first-tier diagnostic method for the genetic screening of fetuses with abnormal ultrasound results, as well as for diagnosing individuals with developmental disabilities or congenital anomalies [29, 30]. In addition, SNP-based CMA is efficient at identifying UPDs, IBD segments and copy number variations. In our recently published paper [31], we identified one case of IBD and two cases of UPD with abnormal phenotypes among a total of 472 prenatal testing samples, suggesting the increased number of reported UPD cases found in prenatal testing due to advances in CMA technology should have come to our attention.

Although clinically relevant UPD phenotypes have been described for different chromosomes (http://upd-tl.com/upd.html), UPD observed in prenatal testing, particularly for rarely reported UPD chromosomes, pose a great challenge for genetic counseling. For example, a complete maternal iUPD of chromosome 4 is very rare, and prenatal observation of this type of UPD has never been reported except once in which a prenatal subject exhibited hUPD4. Recently, Palumbo et al. reported a 10-year-old boy carrying a maternal iUPD of chromosome 4 who presented with a mild intellectual disability and a slight speech delay, but without any dysmorphic features [20], further suggesting that prediction of the clinical impact of rare UPD can be complicated.

The hUPD4 case reported by Kuchinka et al.[17] commenced as a trisomic zygote with nondisjunction during maternal meiosis. In our case, the complete iUPD of chromosome 4 was most likely caused by chromosome segregation errors in meiosisII, however, fertilization of a normal egg by a nullisomic sperm with subsequent salvage of a monosomy by postfertilization duplication of the maternal chromosome 4 might also have resulted in iUPD4.

The pathogenesis of UPD is always determined by several imprinted disorders, such as transient neonatal diabetes mellitus (paternal UPD 6q24), Beckwith-Wiedemann syndrome (paternal UPD 11p15.5), Russell-Silver syndrome (maternal UPD 7p11-12), Temple syndrome (maternal UPD 14q32), Wang syndrome (paternal UPD 14q32), Angelman syndrome (paternal UPD15q11-q13), Prader-Willi syndrome (maternal UPD15q11-q13) and pseudohypoparathyroidism type IB (paternal UPD 20q13) [32]. However, whether there are any important imprinted genes clustered in other chromosomes is currently unclear. In addition, the tissue specific effect of imprinting, in which some genes are expressed from both alleles in the blood but show imprinting in a specific tissue [33–35], further complicate the functional interpretation of UPDs which involves those imprinted genes.

Unmasking of pathogenic autosomal recessive variants in uniparental isodisomy events is another mechanism for the clinical impact of UPD. In the reported maternal UPD4 cases, the diverse clinical impact of UPD4 is most likely due to different homozygous recessive mutations rather than aberrant imprinting. Spena et al. described an iUPD4 case with a fibrinogenemia caused by a 15 kb microdeletion [18]. Middleton et al. reported a complete maternal iUPD4 case with a major predisposition to mood disorders due to active recessive alleles [19]. Cottrell et al. reported that a homozygous recessive mutation in the *SGCB* gene caused limb girdle muscular dystrophy in a maternal UPD4 case [22], and Ding et al. reported that a severe hypodysfibrinogenemia in a UPD4 case is also due to an homozygous mutation [21]. Recently, Aminoff et al. described a maternal UPD4 case with abetalipoproteinemia caused by a homozygous mutation of the *MTTP* gene [36]; Losekot et al. described a polycystic kidney disease in a maternal UPD4 case due to a homozygous mutation in the *PKD2* gene [23]. In addition, there are other published papers that further suggest that the clinical impact of UPD4 is caused by homozygous mutations [37, 38]. Therefore, in our case, we searched for homozygous autosomal recessive variants on chromosome 4 by WES.

In this study, 552 OMIM genes and 131 disease-associated genes were identified in the iUPD4 region involved; however, no pathogenic or likely pathogenic variants were detected for those genes. After filtering using Ingenuity software and bioinformatics analysis, two rare frameshift and two nonsynonymous homozygous variants for four non-disease related genes on chromosome 4 were identified as variants of unknown significance. Although these variants are predicted to have a functional impact, none of these variants are definitively associated with congenital abnormality or inherited disease, and there are no literature reports that these variants cause life-threatening malformations. Therefore, detailed ultrasound fetal morphology and structural scanning was performed regularly in this fetus; we did not find any structural abnormalities, facial dysmorphisms or IUGR. In addition, the couple was also informed that an imprinting effect associated with UPD4 could not be excluded because of the limited understanding of imprinting in specific organisms [34, 35]. A healthy boy was born after the couple was counseled with all of the prenatal diagnostic results. At the last evaluation, the baby showed normal development during his first year of birth.

To the best of our knowledge, this is the first prenatal report of complete maternal iUPD of chromosome 4 without clinical findings utilizing the holistic approach of ultrasound screening, CMA and WES testing. Although no phenotypic abnormalities were observed in this child after his first year of birth, further clinical counseling and long term follow-up is necessary to rule out the possibility of an intellectual disability and/or mood disorder. It should be taken into account that chromosome 4 has been weakly associated with mood disorders [39], and some genes on chromosome 4, such as *RASGEF1B, MAPK10* and *JNK3* [40, 41], are associated with intellectual disability.

In this study, the 1 in 58 high risk of Down syndrome predicted by maternal serum screening was considered as a false positive result by CMA. Whether the complete UPD4 or other UPD event would influence the maternal serum protein secreted and then influence the results of maternal serum screening is unknown. It is interesting to investigate the correlation between UPD events or other comprehensive genetic variants and maternal serum screening results.

In conclusion, due to the limited genetic information and complex effect of UPD and imprinting, prenatal diagnosis of a rare UPD event is complicated. The iUPD4 case that we studied might be benign and completely unrelated to a clinical phenotype. In any event, by combining CMA, WES and ultrasound technology, we were able to provide comprehensive genetic and fetal medicine information for prenatal diagnosis of a rare UPD event.

## Methods

### Ethics and patient

The Ethics Committee of the Third Affiliated Hospital of the Guangzhou Medical University approved this research. Written informed consent was obtained from the couple prior to performing the invasive prenatal diagnosis.

In this study, a 28-year-old gravida 1 para 0 woman was referred to the hospital for a genetic consultation at 20 weeks of gestation because maternal serum screening revealed a 1 in 58 risk for Down syndrome. Her husband was 30 years old. The couple declared that they were non-consanguineous and had no family history of congenital anomalies. An amniotic fluid sample (15 ml) was obtained for karyotyping and CMA testing, and peripheral blood samples from the parents were collected. A fetal cord blood sample was collected at 25 weeks of gestation for further validation.

### Karyotype analysis

For chromosome analysis, 20 metaphase cells from amniotic fluid sample were examined using the G-banding method in situ from two independent cultures and the 400-banding level of chromosome was achieved.

### DNA extraction

Genomic DNA was extracted and purified from uncultured amniocytes, peripheral blood samples and a fetal cord blood sample using a QiagenDNeasy Tissue Kit according to the manufacturer’s instructions (Qiagen, Hilden, Germany).

### SNP-based CMA analysis and data interpretation

High-quality genomic DNA (250 ng) was digested, ligated, PCR amplified, labeled and hybridized to CytoScan 750 K arrays according to the manufacturer's protocol (Affymetrix, Santa Clara, CA, USA). After being washed and stained, the microarrays were scanned using an Affymetrix 7G scanner. The data were analyzed usingAffymetrix Chromosome Analysis Suite (ChAS 2.2, Affymetrix, Santa Clara, CA, USA). Next, 50 probes, a loss-size of 50 kb, a gain-size of 100 kb, and a 5,000 kb UPD region were designated as the analysis thresholds. The locations of the copy number variations (CNVs) and the UPD events were determined based on a human genome assembly from February 2009 (GRCH37/h19).

For data interpretation, the Database of Genomic Variants (DGV), the Database of Chromosome Imbalance and Phenotype in Humans Using Ensemble Resources (DECIPHER), the Clinical Genome Resource (ClinGen), OMIM genes and our lab's in-house database were used to evaluate the CNVs identified in this study. Evaluation of UPD events was performed using the Genomic Oligoarray and SNP array evaluation tool [42] following the guidelines of the Canadian College of Medical Geneticists (CCMG) [43].

### Whole exome sequencing

WES was performed to investigate single nucleotide variants (SNVs) or small insertion/deletions (InDels) in the fetal cord blood. High-quality genomic DNA (100 ng) was amplified in Ion AmpliSeq Exome RDY plates using Ion AmpliSeq HiFi Mix (Ion Torrent, Carlsbad, CA). The resulting 240–280 bp amplicons were treated with FuPa Reagent (Ion Torrent) to partially digest the primers and phosphorylate the amplicons, which were then ligated to Proton adapters and purified according to the manufacturer's instructions (Ion Torrent). Libraries were quantified by quantitative PCR and then loaded onto the Ion Proton platform for high-throughput sequencing. The raw sequencing output data of the Ion sequencer were processed using the Torrent sequence generation algorithm. The standard bioinformatics analysis begins with the raw data generated from the Ion Proton sequencing pipeline. Firstly, TMAP (https://github.com/nh13/TMAP) was used to align reads to the reference sequence. The alignment information was stored in BAM format files. After alignment, the final BAM files were ready for variant calling. SNPs and InDels were all detected using the Torrent Variant Caller (https://github.com/iontorrent/Torrent-Variant-Caller-stable) (TVC) and annotated using Ion Reporter software (IR). Quality control (QC) was required at each stage of the analysis pipeline for the raw data, the alignment and the called variant. The data were aligned and mapped to the NCBI reference genome (GRCH37/h19) and were further analyzed and filtered using NextGene software (SoftGenetics, LLC, PA, USA), the wANNOVAR web server (http://wannovar.usc.edu/) and Ingenuity software (http://www.ingenuity.com/).

### Confirmation of identified variants

WES data were validated by comparison of the SNP information derived from the CMA. Sanger sequencing was used to confirm rare variations identified by WES that were not covered by the probes of the CMA.

## Additional files

Additional file 1: Table S1. Timeframe of gestational weeks for different prenatal analyses. (DOC 30 kb) Additional file 2:Genotyping information of chromosome 4 among the fetus and his parents. (XLSX 769 kb) Additional file 3:Genes located on chromosome 4. (XLSX 415 kb)
