# Supplementary material for: Prenatal diagnosis of complete maternal uniparental isodisomy of chromosome 4 in a fetus without congenital abnormality or inherited disease-associated variations
Source: Mol Cytogenet. 2015 Nov 4;8:85. doi: 10.1186/s13039-015-0190-z (PMC4632482; doi:10.1186/s13039-015-0190-z)
Supplement: Additional file 1: Table S1. — Timeframe of gestational weeks for different prenatal analyses. (DOC 30 kb) [file 13039_2015_190_MOESM1_ESM.doc]

***Supplementary Table 1.*** *Timeframe of gestational weeks for different prenatal analyses*

| Timeframe of gestational weeks   | Tested sample  type | Analysis  method | Results |  | | --- | --- | --- | --- | |
| --- | --- | --- | --- | --- |
| | 16^+2^ | | Peripheral blood | Maternal serum screening | 1 in 58 high risk of Down syndrome | | --- | --- | --- | --- | --- | | 20^+3^ | Amniotic fluid | | Karyotyping; CMA Ultrasound screening | 46XY; iUPD4; No abnormalities detected by ultrasound screening | | 25^+2^ | | Cord blood | WES;  Ultrasound screening | SNV(VOUS, non-disease associated); No abnormalities detected by ultrasound screening | | 30^+1^ | | NA | Ultrasound screening | No abnormalities detected by ultrasound screening | |
